# Inhibitory Activity of Myelin-Associated Glycoprotein on Sensory Neurons Is Largely Independent of NgR1 and NgR2 and Resides within Ig-Like Domains 4 and 5

**DOI:** 10.1371/journal.pone.0005218

**Published:** 2009-04-15

**Authors:** Verena Wörter, Rüdiger Schweigreiter, Bernd Kinzel, Matthias Mueller, Carmen Barske, Günther Böck, Stefan Frentzel, Christine E. Bandtlow

**Affiliations:** 1 Innsbruck Medical University, Biocenter, Division of Neurobiochemistry, Innsbruck, Austria; 2 Novartis Pharma Research, Basel, Switzerland; 3 Innsbruck Medical University, Biocenter, Division of Experimental Pathophysiology and Immunology, Innsbruck, Austria; Ludwig Maximilians University Munich, Germany

## Abstract

Myelin-associated glycoprotein (MAG) is a sialic acid binding Ig-like lectin (Siglec) which has been characterized as potent myelin-derived inhibitor of neurite outgrowth. Two members of the Nogo-receptor (NgR) family, NgR1 and NgR2, have been identified as neuronal binding proteins of MAG. In addition, gangliosides have been proposed to bind to and confer the inhibitory activity of MAG on neurons. In this study, we investigated the individual contribution of NgRs and gangliosides to MAG-mediated inhibition of sensory neurons derived from dorsal root ganglia (DRG) of *ngr1*, *ngr2* or *ngr1/ngr2* deletion mutants. We found no disinhibition of neurite growth in the absence of either NgR1 or NgR2. Sensory neurons deficient for both NgR proteins displayed only a moderate reduction of MAG-mediated inhibition of neurite growth. If treated with Vibrio cholerae neuraminidase (VCN), inhibition by MAG is further attenuated but still not annulled. Thus, disrupting all known protein and ganglioside receptors for MAG in sensory neurons does not fully abolish its inhibitory activity pointing to the existence of as yet unidentified receptors for MAG. Moreover, by employing a variety of protein mutants, we identified the Ig-like domains 4 or 5 of MAG as necessary and sufficient for growth arrest, whereas abolishing MAG's ability to bind to sialic acid did not interfere with its inhibitory activity. These findings provide new insights into the inhibitory function of MAG and suggest similarities but also major differences in MAG inhibition between sensory and central nervous system (CNS) neurons.

## Introduction

MAG/Siglec-4a is expressed in myelinating glia of the central and peripheral nervous system (PNS). It is a member of the Siglecs [1], a sialic acid binding subgroup of the immunoglobulin-superfamily (IgSF). Membrane-bound MAG contains five extracellular Ig-like domains with the N-terminal V-type Ig-domain harboring the sialic acid binding site [2], [3]. Besides its role in long-term maintenance of myelin sheaths and axonal integrity [4], [5], [6], MAG is known to affect axon growth. Originally described to support fiber growth of cultured embryonic and early postnatal neurons [7], [8], MAG was later found to impair fiber growth of mature peripheral and central neurons [9], [10]. Two neuronal proteins of the Nogo-receptor family, NgR1 and NgR2, have been shown to bind MAG with similar affinity and to confer growth arrest [11], [12], [13]. Both receptors are glycosylphosphatidylinositol-linked proteins and require partner proteins for signal transduction. NgR1 forms a tripartite receptor complex with the neurotrophin receptor p75^NTR^ [14], [15] or TROY/TAJ [16], [17], two members of the TNFR superfamily, and the transmembrane leucin rich repeat protein Lingo-1 [18]. However, recent studies question whether TROY can serve as a functional substitute for p75^NTR^ [19], [20]. Membrane spanning constituents of an NgR2 receptor complex have not been identified yet. Besides NgRs, neuronal gangliosides, notably GT1b and GD1a, seem to directly interact with MAG via sialic acid residues that are recognized by MAG's lectin domain and were proposed to act as independent functional MAG receptors [21], [22], [23], [24]. Thus far, resolving the relative contribution of each neuronal MAG receptor constituent in neurite growth inhibitory signaling has been hampered for two reasons. First, for lack of genetic deletion mutants, many experiments on neuronal MAG receptors had to rely on, sometimes ambiguous, biochemical read-out assays. Second, MAG and its receptors were studied in different types of neurons. However, only now is it becoming evident that there are significant differences in the molecular machinery mediating MAG inhibition between various neuronal cell types [20], [24].

To better understand the function and relative contribution of NgR1 and NgR2 in MAG-mediated neurite growth inhibition we analyzed DRG neurons of *ngr1−/−*, *ngr2−/−* and *ngr1/ngr2* double mutant mice in vitro. Sensory neurons are an ideal model system since they are strongly inhibited by MAG and express all identified receptor components [13], [16], [17], [25]. We found that only the combined absence of NgR1/NgR2 and the disruption of gangliosides reduced MAG inhibition, but was still insufficient to fully disinhibit sensory neurons. Furthermore, we found that the inhibitory domains of MAG essential to trigger growth arrest differ between sensory and CNS neurons. Collectively, our findings support a model of MAG inhibition that is cell-type specific and relies on at least three distinct signaling modes.

## Materials and Methods

### Generation of an *ngr2* knock-out mouse strain

For generation of the targeting vector *ngr2* genomic sequences corresponding to intron 1 as well as the 3′ untranslated region (UTR) were amplified and subcloned into the vector pRAY2 (Accession No. U63120). Primers used for amplification of genomic DNA were (for) 5′-TAT AGT CGA CCC TTG GGC TTT GAC CAT GAT C-3′ and (rev) 5′-TCT GGG AGC AAC ACC AGC C-3′ for the amplification of the intron 1 homology region, and (for) 5′-TAT AAT CGA TAA GAC CTC AAA GGC AGC GG-3′ and (rev) 5′-TAT AGC GGA AGC ACG TGA TGG GCG TCC CTT GG-3′ for the amplification of the 3′-UTR homology region. C57Bl/6 mouse ES cell culture (ES cell line Bl6-III) [26], [27] was performed with primary X-ray-inactivated embryonic fibroblasts derived from DR4 mice. ES cells were transfected by electroporation using 12 µg of linearized target plasmid. Transfected ES cells were selected for neomycin resistance using 0.2 mg/ml G418 (Invitrogen #10131-019). Ten days after transfection, G418-resistant ES cell clones were isolated and analyzed by PCR for homologous recombination. Nested PCR reactions were carried out using the Qiagen Taq PCR Master Mix (Qiagen #201445) with the first primer pair: Neo-fw1: 5′-CTT GCC GAA TAT CAT GGT GG-3′; NgRH1-rev1: 5′-TGG CTG GTG TGC TTA CAC TT-3′ and the second primer pair: Neo-5: 5′-CAG GAC ATA GCG TTG GCT AC-3′; NgRH1-F1: 5′-CTG AGG TGC ATC TGC CTG TT-3′. A targeting frequency of 6.25% was observed yielding a total of five targeted ES cell clones. Homologous recombination was confirmed by Southern Blot analysis. Ten micrograms of genomic DNA were digested with 50 units of RsrII and separated on a 0.9% agarose gel. After denaturation the DNA was blotted onto a Hybond N+ membrane (GE Healthcare #RPN203B) followed by UV crosslinking. An NgR2 DNA probe was amplified by PCR using primers NgRH1-SpFw: 5′-AGG CTC AGG TTC TGT TGT CC-3′ and NgRH1-SpRv: 5′-GCT GCC AGA CCT TGG AGT AC-3′. Hybridization with the ^32^P-labeled DNA probe (Rediprime II Random prime labeling kit; GE Healthcare #RPN1633) was performed in Perfect Plus Hybridization buffer (Sigma-Aldrich #H7033) at 65°C overnight. After washing the hybridized membrane, image analysis was performed using a Fuji FLA-5000. Following karyotype analysis ES cells of three targeted clones were injected into Balb/c host blastocysts, which were subsequently transferred into pseudopregnant CB6F1 foster mothers. Chimeric offspring were identified by coat pigmentation (black C57Bl/6 on a white Balb/c background). Black offspring indicated the germline transmission of the targeted ES cells. Genotyping of targeted mice was done via PCR assaying for deletion of exons 2 and 3. Using genomic DNA obtained from tail biopsies two separate PCR reactions were applied in order to discriminate between heterozygous, homozygous and wildtype (wt) mice. PCR primer to detect the wt allele: NgRH1: 5′-TTG TCT GCA GAG CAC CTT CCA C-3′; NgRH1 rev: 5′-TTC TCT GTG TAA CAG CCT TGG G-3′. A 500 bp amplification product is expected. PCR primer to detect the targeted allele: Neo starts: 5′-ATG GGA TCG GCC ATT GAA CAA-3′; NgRH1 rev: 5′-TTC TCT GTG TAA CAG CCT TGG G-3′. A 1.1 kb amplification product is expected. The absence of an NgR2 transcript in brain tissue from homozygous null mice was tested with real-time and reverse transcription PCR. To this end, total RNA was isolated from mouse brain using Tripure Isolation Reagent (Roche #1 667 165) followed by DNaseI treatment. Reverse transcription of RNA was performed with Omniscript RT Kit (Qiagen #205111). PCR was carried out with Taq-polymerase (Roche #1 146 173) and NgR2 specific primers (NgR2for: 5′-TGA CTT GTT CGC GGA CCT GG-3′; NgR2rev: 5′-GAG GAT GGT GAG GCG GCT GA-3′) yielding a product of 181 bp. Real-time PCR was done with a Light Cycler (Roche) using Quantitect SYBR® Green PCR Kit (Qiagen #204143). NgR2 signals were normalized to GAPDH mRNA. The *ngr2* knock-out mouse line was named Bl6-TgH(NgRH1)^143Npa^, according to the guidelines of the *International Committee on Standardized Genetic Nomenclature for Mice*.

The *ngr1−/−* mice are described elsewhere [27]. The absence of NgR1 in knock-out animals was verified by PCR-genotyping of tail biopsies using the following primers: NR3F1: 5′-TCG GCA CAT CAA TGA CTC TCC-3′, NR3R3: 5′-TAT GTA CAC ACA CCT GGT GGC-3′ and bpA2: 5′-TGG GCT CTA TGG CTT CTG AG-3′. A 325 bp amplicon is expected for the wildtype allele and a 210 bp amplicon for the targeted allele.

### DNA constructs

MAGp72_pXM plasmid containing full-length rat L-MAG was a kind gift from Rainer Hillenbrand, Basel, Novartis Institutes for BioMedical Research [28], MOG_pCMV containing full-length human MOG alpha variant was provided by Markus Reindl, Innsbruck Medical University and N-CAM_pBK-CMV containing full-length chicken N-CAM180 was given to us from Jozsef Kiss, Geneva, University Medical Center. The coding sequence of MAG was subcloned in the pKS+ vector using KpnI and BamHI for further cloning steps (MAG_pKS+). The MAG mutant R118A was generated by site-directed mutagenesis using the “QuikChange^TM^ site-directed mutagenesis kit” of Stratagene (#200519) and MAG_pKS+ as template. The primer sequence was: 5′-GGA GGG AAA TAC TAT TTC ***GCA*** GGT GAC CTG GGC GG-3′ and 5′-CC GCC CAG GTC ACC ***TGC*** GAA ATA GTA TTT CCC TCC-3′, changing the original Arg^118^ codon “CGA” to Ala codon “**GCA**”. The mutation was confirmed by sequencing. MAG Ig1–3 was generated by PCR using MAG_pKS+ as template and primers sparing the sequence encoding Ig-like domains 4 and 5 (for: 5′-TAA CCC GGG CTG ATG TGG GCC AAA ATC GGC-3′; rev: 5′- TCC AAC CCG GGT GCA TAC ATG ACG CTG TCG-3′). The MAG replacement mutants rIg4 and rIg5 were constructed from MAG_pKS+ and N-CAM_pBK-CMV plasmid. MAG sequence lacking Ig-like domain 4 or 5 was PCR-amplified using MAG_pKS+ as template and primers containing linkers for HindIII and KasI. The primer sequences were: rIg4 (for: 5′-ATT ***GGC GCC*** CCC ATA ATC CTT CTG GAA TCG CAC-3′; rev: 5′-CCC C***AA GCT T***AG GTG CAT ACA TGA CGC TCA GC-3′; linkers marked) and rIg5 (for: 5′-ATC ***GGC GCC*** CTG ATG TGG GCC AAA ATC-3′; rev: 5′-CCC C***AA GCT T***AG CAA ACT CCA CAG ACA GG-3′; linkers marked). The sequence encoding the Ig4 domain of N-CAM was PCR-amplified using N-CAM_pBK-CMV as template and primers containing linkers for HindIII and KasI (for: 5′-CCC ***AAG CTT*** GAG GAT CAG ATC ACA CTG ACC-3′; rev: 5′-ATC ***GGC GCC*** GAT GGT GTT GCT GGC C-3′; linkers marked). The N-CAM Ig4 amplicon was inserted into the MAG amplicon lacking the sequence for either Ig-like domain 4 or 5. The chimeric cDNAs were finally cloned into the expression vector pBK-CMV (Stratagene #212209) using KpnI and BamHI sites. For generation of chimeric MOG/MAG constructs, the sequence encoding MAG Ig-like domains 4, 5 and 4/5 was PCR-amplified using MAG_pKS+ as template and primers containing StuI and KasI linkers (for Ig4: 5′-***AGG CCT*** TGG AAG CCC ACA GTG AAT-3′; rev Ig4: 5′-***GGC GCC*** GGG AGC AAA CGC CA-3′; for Ig5: 5′-***AGG CCT*** ATA ATC CTT CTG GAA TCG CAC-3′; rev Ig5: 5′-***GGC GCC*** TCG GTG TGC TCC-3′; linkers marked). Amplicons were cloned into MOG_pKS+ cut with StuI and KasI thus inserting the MAG domains between Arg^130^ and Asp^131^ of MOG. Chimeric MOG/N-CAM was generated by PCR-amplifying the sequence encoding the Ig4 domain of N-CAM using N-CAM_pBK-CMV as template and primers with StuI and KasI linkers (for: 5′-CCT ***AGG CCT*** GAG GAT CAG ATC ACA CTG ACC-3′; rev: 5′-ATC ***GGC GCC*** GAT GGT GTT GCT GGC C-3′; linkers marked) and inserting the amplicon into MOG_pKS+ cut with StuI and KasI. The MOG/MAG and MOG/N-CAM constructs without the signal peptide were subcloned in a modified pAPtag-5 vector (GenHunter #Q202) containing an N-terminal V5-tag.

### CHO-K1 cell lines

CHO-K1 cells were grown as described previously [29]. To generate CHO-K1 clones stably expressing wildtype or mutant MAG, cells were transfected with respective constructs using Lipofectamine 2000 (Invitrogen #11668-019) and selected with 1 mg/ml G418 (pBK-CMV-constructs; Invitrogen #11811-031) or 200 µg/ml zeocin (pAPtag-5-constructs; Invitrogen #45-043). Transfected cells were subjected two to three times to fluorescence-activated cell sorting (FACS) using the monoclonal anti-MAG 513 antibody (Millipore #MAB1567) for MAG R118A, MAG Ig1–3, MAG rIg4 and MAG rIg5, the monoclonal anti-V5 antibody (Invitrogen #R960-25) for the MOG/MAG and MOG/N-CAM chimeric proteins and the monoclonal anti-MOG (clone 8.15-C5; kind gift from Markus Reindl, Innsbruck Medical University) for MOG transfected cells. Clonal cell lines were obtained by limiting dilution of the sorted cell populations. Aliquots of a dilution containing in theory 1 cell/100 µl were seeded in a 96-well plate. Screening for positive clones was done with live cell surface immunofluorescent staining. Clones with highest expression level were chosen for neurite outgrowth assays.

### Immunocytochemistry

For live-staining CHO cells expressing MAG or MAG-mutants were seeded onto glass cover slips. After overnight culture they were washed once with PBS and incubated with 10 µg/ml anti-MAG 513 antibody or 1 µg/ml anti-V5 antibody or 2.5 µg/ml anti-MOG (clone 8.15-C5) for 25 min at room temperature. Following fixation with 4% (w/v) paraformaldehyde and 5% (w/v) sucrose in PBS cells were incubated for one hour at room temperature with a Rhodamine-labeled goat anti-mouse IgG (1∶700; Millipore #AP124R). The coverslips were mounted (Fluorescence mounting medium; Dako #S3023) and pictures were taken with a Zeiss Axioplan2 microscope equipped with a spot camera (RT-Slider 2.3.1, Visitron Systems, Puchheim, Germany).

### Sensory neurons

Preparation of sensory neurons from DRG was described previously [30]. Briefly, ganglia were obtained from P7-P9 wildtype or transgenic mice. Ganglia were digested two times, 30 min each, at 37°C with 0.09 mg/ml liberase blendzyme 1 (Roche #11 988 409 001) in DMEM, washed twice with PBS and incubated for another 30 min with 0.05% (w/v) trypsin (Invitrogen #15090). After trituration cells were spun through DMEM/3.5% (w/v) BSA and resuspended in complete medium [31]. Sensory neurons were plated in complete medium onto monolayers of CHO-K1 cells.

### Neurite outgrowth assay and statistical analysis

Neurite outgrowth assays on confluent monolayers of parental CHO-K1 cells or CHO cells overexpressing wildtype MAG, mutant MAG, MOG/MAG or MOG/N-CAM chimeras was performed as described previously [32]. Briefly, approximately 6000 sensory neurons were added to the CHO-K1 monolayers in complete medium. Where indicated Vibrio cholerae neuraminidase (VCN; Sigma-Aldrich #N7885) was added at the indicated concentration one hour after plating the neurons. Specificity of VCN treatment was revealed as described in Method S1. Following an incubation period of approximately 20 hours cells were fixed and neurons were stained for better visualization with anti-GAP43 as described [32]. The coverslips were mounted and fluorescent pictures from isolated neurons were taken in a systematic manner with a Zeiss Axioplan2 microscope equipped with a spot camera (RT-Slider 2.3.1, Visitron Systems, Puchheim, Germany). Neurite length was determined by measuring the maximal distance from the center of the cell body to the furthest neurite from digitized images of approximately 50 individual neurons per experiment and condition using MetaVue 5.0r3 software (Universal Imaging Corporation, PA, USA). The mean and SEM of neurite-bearing cells were calculated from at least three to seven independent experiments. Data were analyzed by one-way ANOVA, followed by Tukey *post hoc* analysis with SPSS 15.0.1 2006 for Windows (SPSS, IL, USA). Error bars indicate SEM.

### Western Blotting

Cells were lysed in 50 mM Tris-HCl pH 7.5, 150 mM NaCl, 0.1% (w/v) SDS, 1% (v/v) NP40, 10 µg/ml aprotinin, 5 µg/ml leupeptin, 1 µg/ml pepstatin and 174 µg/ml phenylmethylsulfonyl fluoride (PMSF). Lysates were sonicated for 10 sec and centrifuged for 15 min at 4°C at 21.000×g. Protein concentration was determined using BioRad Protein Assay (#500-0006), β-mercaptoethanol was added and lysates were incubated at 95°C for 5 min before they were subjected to SDS-PAGE. Proteins were transferred onto polyvinylidene difluoride membrane (Millipore, Immobilon-P, #IPVH00010) and blocking was carried out with 3% (w/v) gelatine (hydrolysed gelatine #17079/1; Naumann – Gelatine und Leim GmbH, Memmingen, Germany) in TBS/0.1% (v/v) Tween-20 for 1 hour. Proteins were detected with antibodies as indicated: 0.2 µg/ml polyclonal goat anti-MAG (L-20; Santa Cruz #sc-9543), 0.25 µg/ml monoclonal mouse anti-MOG (clone 8.15-C5), 0.107 µg/ml monoclonal mouse anti-V5 (Invitrogen #R960-25), 0.29 µg/ml polyclonal goat anti-Flotillin-1 (K-19; Santa Cruz #sc-16640), 0.1 µg/ml monoclonal mouse anti-α-Tubulin (DM1A; Sigma-Aldrich #T 9026) overnight at 4°C in 1.5% (w/v) gelatine in TBS/0.1% (v/v) Tween-20. After three washes with TBS/0.1% (v/v) Tween-20, membranes were incubated with the HRP-conjugated secondary antibodies rabbit anti-goat IgG (1∶14.000; Pierce #31402) or goat anti-mouse IgG (1∶20.000; Pierce #31432) for one hour at room temperature in 1.5% (w/v) gelatine in TBS/0.1 % (v/v) Tween-20. After three washing steps HRP activity was detected with ECL-Plus Western blotting detection reagent (GE Healthcare #RPN 2132) using a Typhoon 9410 scanner (GE Healthcare). Glycosylation patterns were revealed as described in Method S2.

## Results

### Generation of *ngr* mutant mouse strains

**Figure 1:**
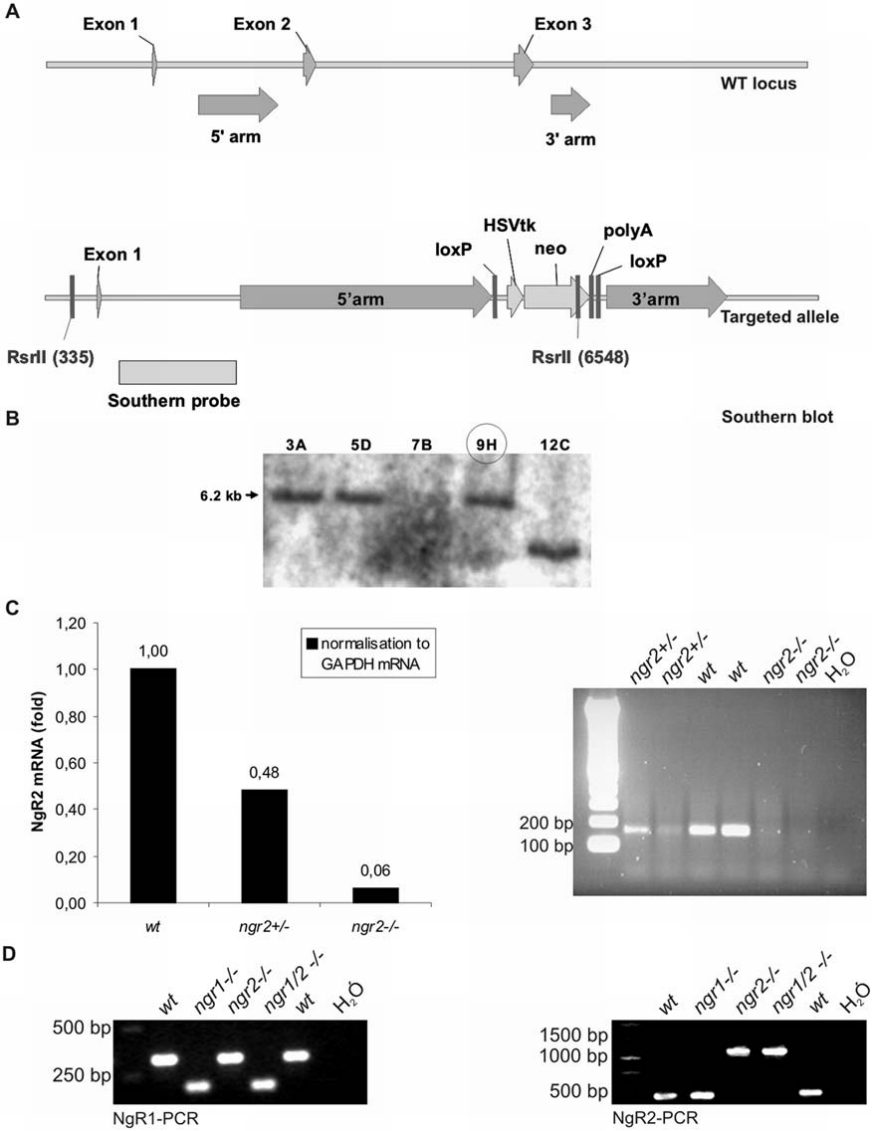
Generation of *ngr2* deletion mutant mice. (A) Targeting strategy for the *ngr2* mutant. Genomic sequences in intron 1 and 3′UTR of the *ngr2* gene were used as targeting sites. (B) Southern analysis of ES cell clones; the expected signal for a targeted clone is at 6.2 kb. Germline transmission after blastocyst injection was obtained for ES cell clone 9H (circled). (C) Real-time-PCR (left) and reverse transcription PCR (right) for NgR2 with mouse brain cDNA confirm the absence of NgR2 in −/− animals. (D) Genotyping of mice using genomic DNA from tail biopsies of wt, *ngr1−/−*, *ngr2−/−* and *ngr1/2−/−* animals. HSVtk, Herpes simplex virus thymidine kinase gene; neo, neomycin resistance gene; polyA, polyadenylation site.

Inactivation of the murine *ngr2* gene was achieved by targeted disruption of exons 2 to 3 with a strategy depicted in Figure 1A. Except for exon 1, which encodes only 10 amino acids, the entire coding sequence was deleted and the mutation was therefore expected to be a null allele. The targeted allele was obtained in C57Bl/6 ES cells (B16-III cell line; [26]) as confirmed by Southern Blotting of ES cell genomic DNA (Fig. 1B). ES cells of three targeted clones were injected into Balb/c host blastocysts and germline transmission was achieved with one clone. Real-time and reverse transcription PCR with brain cDNA of homozygous null mice confirmed the absence of the NgR2 transcript (Fig. 1C). At genomic level, the mutant allele was detected by PCR-based genotyping of tail biopsies (Fig. 1D). Expression of NgR1 or other known receptor components conferring MAG responsiveness such as p75^NTR^ or TROY/TAJ were unaltered in cultured sensory neurons (data not shown). Heterozygous *ngr2* mice appeared phenotypically normal and fertile, and intercrosses between heterozygous mice yielded homozygous *ngr2* knock-out animals at a normal Mendelian frequency. Similar to *ngr1* deletion mutants [27], homozygous adult *ngr2* null mice appeared healthy, and histological examination showed no obvious abnormalities in the brain, liver or heart (not shown). *ngr1/2* double null mice were generated by cross-breeding *ngr1* and *ngr2* deletion mutants and the absence of both the *ngr1* and *ngr2* wildtype allele was demonstrated by PCR-based genotyping of tail biopsies (Fig. 1D). *ngr1/2* double null mice are viable and fertile and morphologically indistinguishable from wildtype animals.

### Absence of NgR1 or NgR2 alone does not attenuate MAG inhibition of sensory neurons

**Figure 2:**
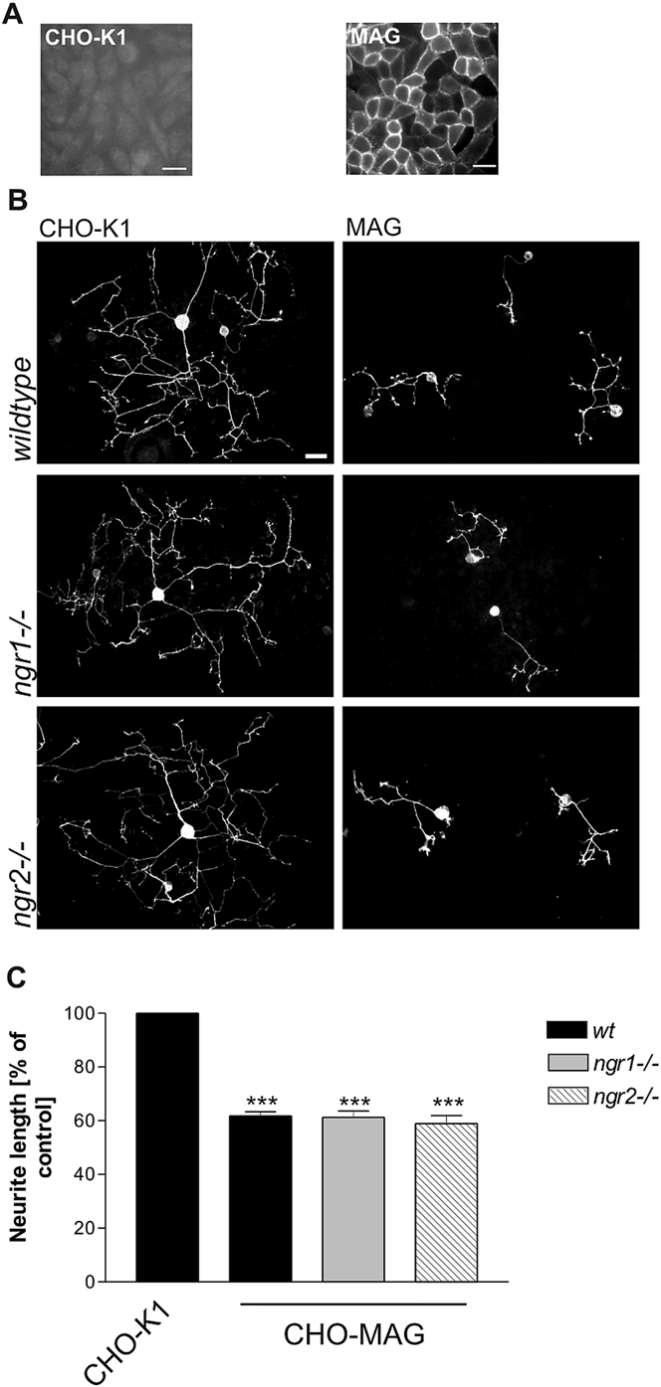
MAG-mediated inhibition of neurite outgrowth of *ngr1−/−* and *ngr2−/−* sensory neurons. (A) A clonal CHO-K1 cell line stably expressing MAG was raised and characterized. Live anti-MAG immunostaining (parental CHO-K1 versus MAG overexpressing clone) demonstrated surface expression of MAG. Scale bar 20 µm. (B) Photomicrographs show GAP-43-positive DRG neurons from wildtype, *ngr1−/−* and *ngr2−/−* mice after a culture period of 20 hours on CHO control or CHO-MAG substrates. Neurite extension of all genotypes is markedly reduced on MAG expressing CHO-K1 cells. Scale bar 40 µm. (C) Quantification of neurite length of DRG neurons from wildtype, *ngr1−/−*, or *ngr2−/−* mice grown on MAG-expressing CHO cells. CHO-K1 cells served as the control substrate. Graph shows % neurite length on the different substrates±SEM. At least five separate experiments were examined for each experimental condition, with 100–150 neurons measured for each substrate. *** represents *P*≤0.001 compared to CHO-K1, one-way analysis of variance with post-hoc Tukey test.

Previous studies showed that MAG transduces its repulsive signals by binding to NgR1 [11], [12] or NgR2 [13]. Because both receptors are abundantly expressed on DRG neurons [13], [25] we were interested to study their relative contribution to MAG inhibition in a mouse genetic approach. In order to present MAG to neurons in a physiological manner, we used a CHO-K1 cell line that expressed significant levels of MAG on the cell surface as revealed by live-staining (Fig. 2A) and tested its ability to modulate neurite outgrowth of postnatal sensory neurons from mice deficient for NgR1 or NgR2. As reported previously [10], sensory neurons grown on a CHO-MAG substrate have shorter and less branched neurites compared with those grown on a CHO control substrate (Fig. 2B). Quantitative analysis revealed that neurite length of wildtype murine sensory neurons is significantly reduced by approximately 40% on CHO-MAG cells compared to parental CHO-K1 cells (Fig. 2B,C). In agreement with recent reports [27], [33] we found no difference in MAG inhibition between wildtype neurons and *ngr1* null neurons (Fig. 2B,C). Likewise, neurite extension of sensory neurons from *ngr2−/−* mice was as potently inhibited as that of wildtype or *ngr1−/−* animals (Fig. 2B,C). Thus, sensory neurons of wildtype and *ngr* mutant genotype were indistinguishable in neurite extension on control CHO cells and equally inhibited on CHO-MAG cells (Fig. 2C).

Together these observations imply that deletion of NgR1 or NgR2 alone is not sufficient to render sensory neurons insensitive to MAG inhibition.

### Additive, but incomplete, attenuation of MAG inhibition upon combined deletion of NgR1 and NgR2 and neuraminidase treatment

**Figure 3:**
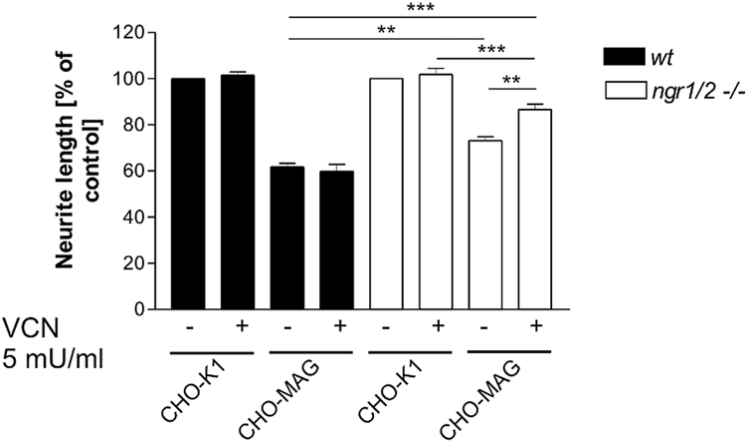
MAG-mediated inhibition of neurite outgrowth of *ngr1/2−/−* sensory neurons with and without VCN treatment. Sensory neurons derived from wildtype or *ngr1/2−/−* mice were seeded onto parental and MAG expressing CHO-K1 cells. Treatment with 5 mU/ml Vibrio cholerae neuraminidase (VCN) was started one hour after plating. Cells were fixed approx. 20 hours after plating. Graph shows % neurite length±SEM of wildtype and *ngr1/ngr2* double knock-out neurons on the different substrates. At least three separate experiments were examined for each experimental condition, with 100–150 neurons measured for each substrate. *** represents *P*≤0.001, ** represents *P*≤0.01 one-way analysis of variance with post-hoc Tukey test.

Given the high degree of homology between NgR1 and NgR2 we hypothesized that the lack of a robust phenotype in the neurite outgrowth response to MAG from neurons deficient for either NgR1 or NgR2 was attributed to functional redundancy between the two receptor constituents. To test this hypothesis, we analysed sensory neurons from *ngr1/ngr2* double knock-out mice for their MAG responsiveness. Surprisingly, in comparison to wildtype or single mutant neurons, combined deletion of NgR1 and NgR2 resulted in only a moderate, albeit significant, release of inhibition of approximately 33% (Fig. 3). No difference was observed between wildtype and double null neurons on control CHO cell substrate. This result demonstrates a partial functional redundancy between NgR1 and NgR2 in sensory neurons with regard to inhibitory MAG signaling, and implies that additional neuronal constituents account for the residual response.

Because recent findings suggest that gangliosides, notably GT1b and GD1a, can act as functional MAG receptors on neurons in a sialic acid dependent manner [21], [22], [23], [24], we examined whether disrupting gangliosides on *ngr1/ngr2* double null neurons would result in full release of MAG inhibition. For this purpose we cultured wildtype and mutant sensory neurons in the presence of different concentrations of vibrio cholerae neuraminidase (VCN). This enzyme removes terminal sialic acid residues from sialylated cell surface molecules including gangliosides and was shown to render sensory neurons less responsive to MAG [24]. In control experiments we demonstrated the effectiveness and specificity of VCN in our neuronal cell culture by showing that VCN treatment caused an increase in the level of membrane monosialylated GM1 as determined by cholera toxin binding (Fig. S1). To our surprise, in the presence of 5 mU/ml of VCN MAG inhibition of double null sensory neurons was attenuated from 73% (untreated) to only 86% (VCN-treated) (Fig. 3). Also at higher VCN concentrations the inhibitory response could not be further reduced (data not shown), indicating that the VCN concentration was at saturation. Thus, the combined absence of NgR1, NgR2 and sialic acid bearing gangliosides diminished MAG inhibition of sensory neurons by only 65%. Consistent with a previous study using wildtype rat retinal ganglion cells [20], we found that the presence of VCN did not result in a significant MAG disinhibition of wildtype sensory neurons (Fig. 3), presumably due to functional compensation by the NgR receptors.

Together, these results indicate that both NgR proteins and gangliosides are involved in MAG inhibition, but are not the only molecules to trigger MAG-mediated growth arrest of sensory neurons.

### Construction and functional analysis of MAG deletion and replacement mutants

**Figure 4:**
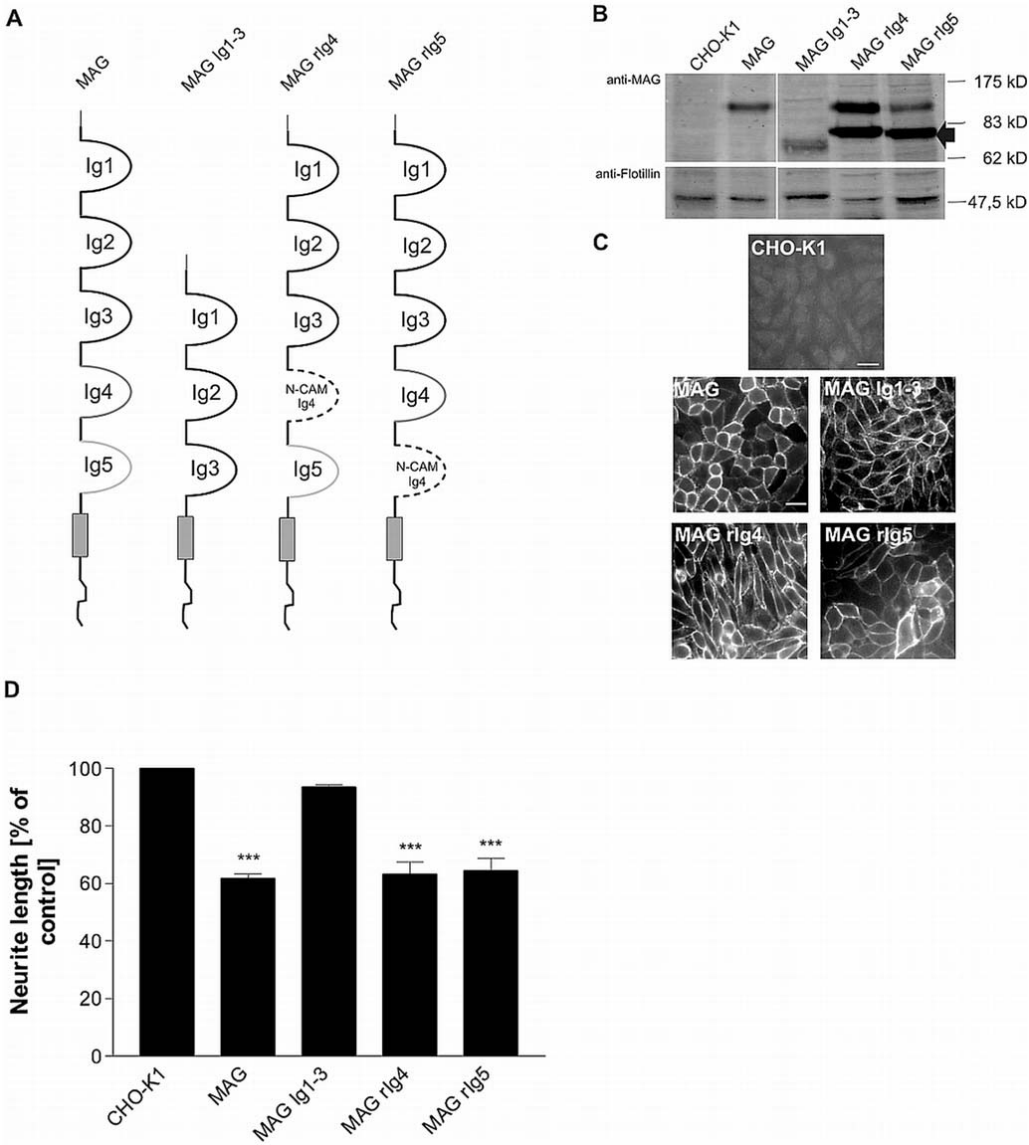
Inhibition of neurite outgrowth by MAG mutants lacking the Ig-like domains 4 and/or 5. (A) Domain composition of the full-length MAG protein and chimeric proteins used to transfect CHO cells in this study. Specific regions within the MAG ectodomain were either deleted (MAG Ig1–3) or replaced by the N-CAM Ig4 domain (dashed line). (B) Western Blot analysis of clonal cell lines expressing MAG mutants (arrow points to not fully glycosylated form; see Fig. S2). Ten micrograms of total protein lysate were loaded in each lane. Goat anti-MAG antibody was used to detect MAG; to reveal equal protein load, blots were incubated with anti-flotillin antibody. (C) Cell surface expression of full-length MAG and chimeric MAG proteins by transfected CHO cells after FACS and clonal selection as revealed by live-staining with anti-MAG antibody. Scale bar 20 µm. (D) Analysis of neurite length of wildtype DRG neurons grown on CHO cell monolayers expressing full-length or chimeric forms of MAG. Parental CHO cells served as the control substrate. Graph shows % neurite length on the different substrates±SEM. Three to seven separate experiments were examined for each experimental condition, with 50 to 100 neurons measured for each substrate. *** represents *P*≤0.001 compared to CHO-K1, **p≤0.01, *p≤0.05 one-way analysis of variance with post-hoc Tukey test.

Given significant differences in the configuration of the MAG receptor complex between sensory neurons and cerebellar granule cells [20], [24] we next asked whether cell-type specific differences of MAG inhibition exist also at the ligand level. Previous reports by Filbin's lab suggested that the neurite inhibitory domain on MAG resides within the Ig-like domains 4 to 5 since mutant MAG comprising Ig1–3 (MAG Ig1–3) does not inhibit neurite outgrowth [3]. More recently, the inhibitory site on MAG was mapped to Ig-like domain 5 for cerebellar granule neurons (CGN) [34]. To test if the same or other domains are responsible for mediating MAG's growth inhibiting activity for murine DRG neurons, we created several deletion and chimeric mutants as shown in Fig 4A. To determine whether the deletion and chimeric proteins were expressed in cells and translocated to the cell surface we produced CHO cell lines expressing these mutant MAG constructs. Live stainings of the transfected cell lines as is shown in Fig. 4C revealed that all mutant proteins were expressed on the cell surface of CHO cells, suggesting that none of these proteins was impaired in expression, overall folding or translocation to the cell surface. Independent stable lines were obtained following FACS and serial subcloning. Clones expressing near-equivalent amounts of full-length and mutant forms of MAG (Fig. 4B) were used for neurite outgrowth experiments described below.

### MAG Ig4 or Ig5 is necessary for inhibition of sensory neurons

Quantification of neurite outgrowth of wildtype sensory neurons on CHO-MAG Ig1–3 cells revealed that neurite elongation is not impaired (Fig. 4D) and does statistically not differ from neurons grown on CHO control cells. This finding is in agreement with an identical approach using CGN [34] and suggests that the inhibitory region of MAG for sensory neurons lies within the Ig-like domains 4 and/or 5. To examine which of the two Ig domains is responsible for mediating the growth inhibiting activity, we replaced MAG Ig4 or Ig5 by the Ig4 domain of the chicken N-CAM molecule, a related molecule that lacks inhibitory activity. Although the N-CAM Ig4 domain is slightly smaller than the MAG Ig domains (74 aa versus 86 aa for Ig4 and 96 aa for Ig5), the resulting chimeras MAG rIg4 and MAG rIg5 retained the domain organization of the parental MAG molecule.

As observed with CGN [34], neurite length of DRG neurons was found to be equally inhibited on CHO-MAG rIg4 as on wildtype MAG (Fig. 4D). To our surprise, however, sensory neurons were also fully inhibited on CHO-MAG rIg5 (Fig. 4D), despite the fact that MAG rIg5 expression was lower in comparison to wildtype MAG or MAG rIg4 (Fig. 4B). This result clearly demonstrates a redundancy between the Ig-like domains 4 and 5. If both are deleted the inhibitory activity of MAG is abolished, but the presence of either domain 4 or 5 is sufficient to confer full inhibition. These findings add significantly to the concept of cell-type specific mechanisms of MAG inhibition, in particular when comparing sensory and cerebellar granule neurons.

### Introduction of MAG Ig4 or Ig5 is sufficient to convert a non-inhibitory molecule into a neurite outgrowth inhibitor

**Figure 5:**
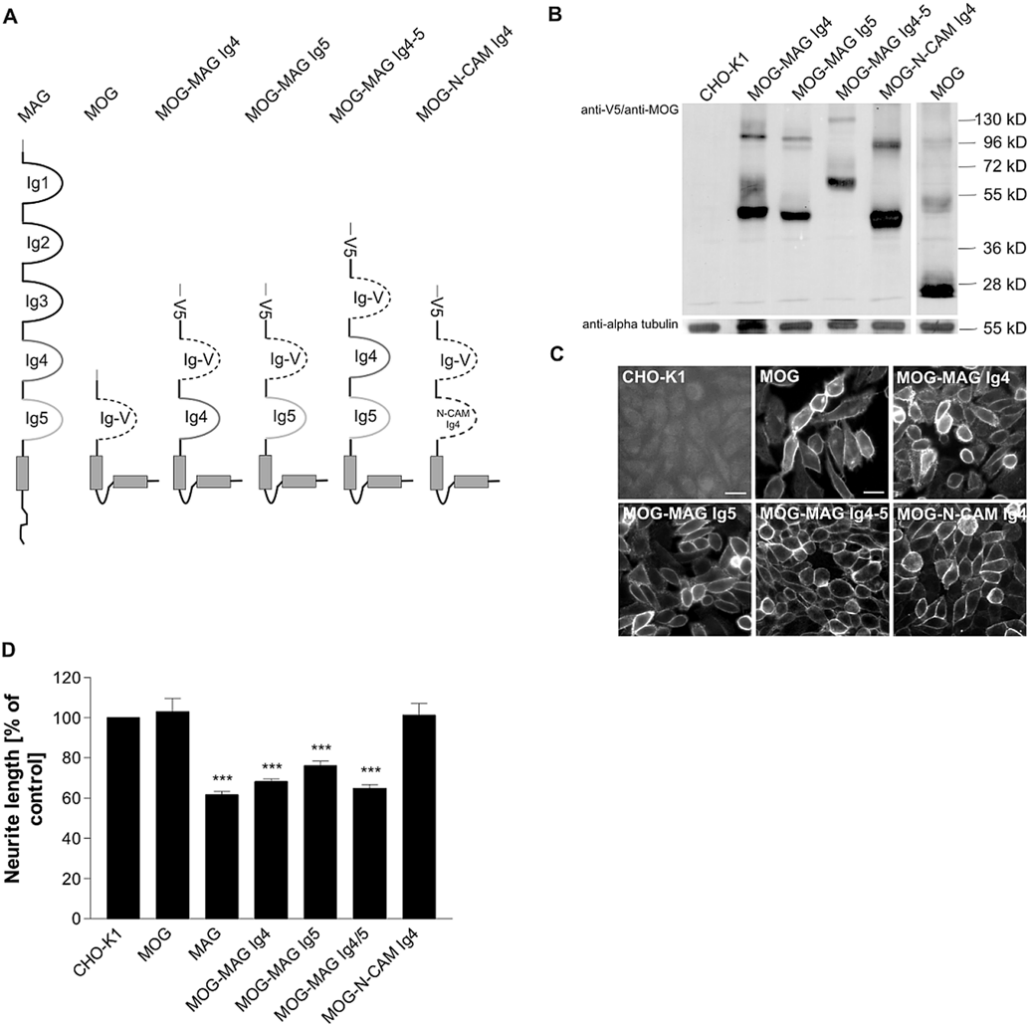
Inhibition of neurite outgrowth by chimeric MOG-MAG molecules. (A) MOG-MAG chimeras, in which either one or two domains of MAG (grey lines) were fused to the MOG Ig-V domain. MOG-N-CAM Ig4 indicates the fusion of the Ig4 domain of the N-CAM molecule to the MOG Ig-V domain. All chimeric proteins have a signal peptide (SP) and carry the cytoplasmic part of MOG along with a V5-tag at their N-terminus. Stable CHO-K1 clones expressing either of these chimeras were generated and characterized by (B) Western Blotting and (C) live anti-V5 immunostaining demonstrating surface expression of each chimera. Scale bar 20 µm. Respective analysis of a MOG expressing CHO-K1 clone was done using an anti-MOG antibody. α-tubulin served as loading control for Western Blots. (D) Analysis of neurite length of wildtype DRG neurons grown on CHO cell monolayers expressing full-length or chimeric forms of MAG. Parental CHO cells served as the control substrate. Graph shows % neurite length on the different substrates±SEM. Three to seven separate experiments were examined for each experimental condition, with 50 to 100 neurons measured for each substrate. *** represents *P*≤0.001 compared to CHO-K1, **p≤0.01, *p≤0.05 one-way analysis of variance with post-hoc Tukey test.

To test whether the Ig4 and/or Ig5 domains of MAG are sufficient to inhibit neurite growth as single domains, we created a second set of chimeras, where Ig4 and/or Ig5 were fused to the Ig domain of MOG (myelin oligodendrocyte glycoprotein; Fig. 5A). MOG is a CNS myelin specific cell-adhesion molecule with no inhibitory activity on postnatal DRG (Fig. 5D). We placed Ig-like domains 4, 5 and 4/5 of MAG between the transmembrane domain and Ig-V of MOG giving rise to chimeric MOG-MAG proteins MOG-MAG Ig4, MOG-MAG Ig5 and MOG-MAG Ig4–5, respectively (Fig. 5A). CHO cells transfected with the various constructs expressed the chimeric molecules on the cell surface (Fig. 5C) and clonal cell lines expressing comparable levels of wildtype MAG and chimeras (Fig. 5B) were used for neurite growth analysis. Results of neurite outgrowth assays obtained with these gain-of-function constructs are shown in Fig. 5D. While growth of sensory neurons was not impaired on MOG, we found that MOG-MAG Ig4 and MOG-MAG Ig4/5 achieved virtually the same inhibitory activity as full-length MAG (Fig. 5D). Neurite growth was significantly, albeit slightly less inhibited on MOG-MAG Ig5 (Fig. 5D), suggesting that the Ig-like domain 5 of MAG has somewhat weaker inhibitory potential than the Ig-like domain 4, at least in the structural context of the MOG molecule.

Although these results already suggest that the presence of each single Ig domain is sufficient to convey inhibition, we wanted to exclude that these effects are induced by the altered structure of the MOG protein backbone. As control, we constructed a MOG-N-CAM chimera, MOG-N-CAM Ig4, with the Ig4 domain of chicken N-CAM inserted into MOG (Fig. 5A). Chimeric MOG-N-CAM Ig4 failed to inhibit neurite outgrowth suggesting that the observed effects were not due to a possible conformational change of MOG protein (Fig. 5D). In essence, these data fully confirm our findings obtained with the MAG replacement mutants.

Taken together, for sensory neurons the inhibitory region of MAG seems to cover the Ig-like domains 4 and 5; either domain - 4 or 5 - is necessary and sufficient to confer full inhibitory activity on neurite outgrowth. This finding is in marked contrast to cerebellar granule neurons which are inhibited by MAG's Ig-like domain 5, but not 4 [34], and demonstrates that cell-type specific mechanisms of MAG inhibition exist not only at the receptor, but also at the ligand level.

### Removal of the sialic acid binding site does not attenuate MAG inhibition

**Figure 6:**
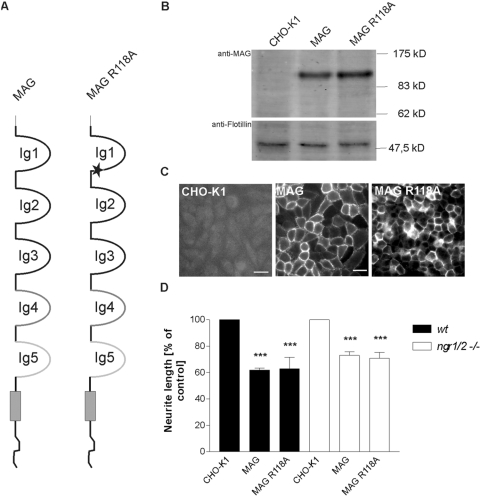
Inhibition of neurite outgrowth by a MAG mutant lacking the sialic acid binding site. (A) The name MAG R118A refers to a point-mutation within the sialic-acid binding domain of MAG (asterisk), that abolishes MAG binding to specific gangliosides. (B) Western Blot analysis of a CHO clone stably expressing MAG R118A. Ten micrograms of total protein lysate were loaded for each lane. Goat anti-MAG antibody was used to detect MAG; to reveal equal protein load, blots were incubated with anti-flotillin antibody. (C) Cell surface expression of full-length MAG and MAG R118A proteins by transfected CHO cells after FACS and clonal selection as revealed by live-staining with anti-MAG antibody. Scale bar 20 µm. (D) Analysis of neurite length of wildtype and *ngr1/ngr2* double knock-out DRG neurons grown on CHO cell monolayers expressing full-length MAG or MAG R118A. Parental CHO cells served as the control substrate. Graph shows % neurite length on the different substrates±SEM. Three separate experiments were examined for each experimental condition, with 50 to 100 neurons measured for each substrate. *** represents P≤0.001 compared to CHO-K1, one-way analysis of variance with post-hoc Tukey test.

A characteristic feature of MAG is its sugar binding ability and it was investigated early on whether the lectin activity of MAG is related to its inhibitory effect on neurons. Previous studies showed that Arg^118^ in Ig domain 1 is crucial for sialic acid binding of MAG [3], but that the sialic acid binding ability of MAG is not necessary for inhibition [3]. The latter experiments were carried out with CGN and we wondered whether sialic acid binding of MAG is of any significance for conferring inhibitory activity on sensory neurons. By site-directed mutagenesis we constructed a MAG mutant that had Arg^118^ replaced with Ala, termed MAG R118A (Fig. 6A). A CHO clone that stably expressed MAG R118A on the cell surface (Fig. 6B, C) was used as a substrate for sensory neurons. No difference in the neurons' responsiveness to the MAG mutant in comparison to wildtype MAG was observed neither with wildtype nor *ngr1/ngr2* double knock-out neurons (Fig. 6D), confirming that the sialic acid binding ability of MAG is not necessary for its inhibitory activity on neurons.

## Discussion

In the present study we investigated the distinct contribution of the known protein and lipid-type MAG receptors to neurite outgrowth inhibition in DRG derived sensory neurons. Using a direct mouse genetic approach we found that neither NgR1 nor NgR2 play a prominent role in transducing MAG's inhibitory growth effects. Likewise, removal of cell surface terminal sialic acids alone did not attenuate MAG inhibition. Remarkably, even the combined loss of NgRs and terminal sialic acids was not sufficient to fully overcome MAG inhibition. Based on these observations we suggest that MAG can signal via other, yet unidentified receptor molecules. In addition, we provide evidence that cell-type specific differences of MAG inhibition exist not only at receptor level, as suggested by previous studies, but also at ligand level.

MAG is one of the best characterized myelin-associated proteins found in both the PNS and CNS [35]. Prior studies demonstrated that MAG not only contributes to the long-term maintenance of axon-myelin integrity [4], [5], [6], but impairs axon growth. MAG effectively restricts neurite outgrowth of late postnatal neurons in vitro [9], [10] and appears to inhibit fiber regeneration in the injured peripheral [36] and central [37] adult nervous system in vivo. Characterizing the neuronal receptor complex that mediates inhibitory MAG signaling revealed that MAG binds independently to different receptors, including NgR1 [11], [12], [13] and NgR2 [13], two GPI-anchored receptor proteins, as well as to GT1b and GD1a [21], [22], two major neuronal gangliosides (sialylation was shown to be mandatory for MAG binding). Based on biochemical and immunological experiments, all four interactions were proposed to be relevant for MAG inhibition, but the relative contribution of each interaction remained elusive. The functional significance of NgR1 has recently been challenged, since the absence of NgR1 on sensory neurons or cerebellar granule cells did not lead to disinhibition of neurite growth on preparations of total myelin [27] or on MAG presenting CHO-K1 cells [20], [33]. Since MAG binds with similar affinity to the structurally related NgR2 protein [13], the role of NgR1 might have been compensated partly by that of NgR2 in *ngr1* knock-out mice. Alternatively, the selective interaction of MAG with gangliosides could compensate for the loss of NgR1. Hence, the generation of *ngr2* and *ngr1/2* deletion mutant mice provides unique animal models that permit investigation into the relative contribution of these receptors in MAG-dependent neurite growth inhibition. As prior studies noted for NgR1 [20], [33], absence of NgR2 alone was not sufficient to release sensory neurons from MAG inhibition. Also the enzymatic removal of terminal sialic acids did not disinhibit sensory neurons as was reported recently for rat retinal ganglion cells [20]. If NgR1 and NgR2 are functionally redundant, neurons of *ngr1/2* double knock-out mice would be expected to be significantly less inhibited by MAG. However, inhibition of sensory neurons was only moderately released, suggesting that NgR proteins play only a minor role as functional inhibitory MAG receptors. We hypothesized that compensation at the level of gangliosides might account for these unexpected findings because MAG independently binds to the gangliosides GT1b and GD1a. Consistent with this idea VCN further alleviated the inhibitory effect of MAG, but failed to annul it. These data not only suggest that MAG uses independent pathways to signal growth inhibition, but in addition support the existence of as yet unidentified functional receptors on sensory neurons which are independent from NgRs and terminal sialic acids. Precisely which additional receptor components may play a role for MAG inhibition remains to be elucidated. Although the neurotrophin receptor p75^NTR^ has been reported to be involved in inhibitory MAG signaling [14], [15], [20], [38], the mechanism seems to be an indirect one, possibly by forming a receptor complex with NgR1, because MAG does not physically interact with p75^NTR^ [38].

Interestingly, further in vitro studies on the role of p75^NTR^ in MAG inhibition pointed to cell-type specific differences in the molecular machinery of MAG signaling. While DRG derived sensory neurons deficient for p75^NTR^ are partially released from MAG inhibition, no disinhibition could be observed for CGNs lacking p75^NTR^ [20]. Such cell-type specificities are supported by a recent study using pharmacological agents to determine the roles of different neuronal MAG receptors [24]. These findings prompted us to investigate whether cell-type specific variations in MAG signaling on the receptor side are reflected at the ligand level. MAG as ligand has been well characterized with regard to inhibition of CGN. Although MAG, as lectin, binds with high specificity to α2,3-linked sialic acid residues, this feature does not seem to play a role for conferring inhibitory activity as demonstrated with a MAG mutant, MAG R118A, that has lost its affinity to sialic acid but is still inhibitory to CGN [3]. Furthermore, the inhibitory region on MAG was mapped to the Ig-like domain 5 using CGN as responsive neurons. Replacing the Ig-like domain 5 of sialoadhesin, like MAG a member of the siglec family, with the Ig-like domain 5 of MAG was shown to be necessary and sufficient to convert non-inhibitory sialoadhesin into an inhibitory molecule with similar potency as wildtype MAG [34]. To characterize MAG as ligand for sensory neurons, we tested MAG R118 for its inhibitory potential. We did not see disinhibition with MAG R118A indicating that sialic acid binding of MAG is not necessary for inhibiting sensory neurons, thus confirming data obtained with CGN. However, we did find a significant difference to CGN inhibition with regard to the inhibition site of MAG. In contrast to results obtained with CGN [34], we localized the inhibitory region of MAG, using sensory neurons and two types of chimeric proteins, over both Ig-like domains 4 and 5. These data clearly indicate that the growth inhibitory site on MAG is defined via the neuronal cell-type MAG is interacting with. Furthermore, these results illustrate that variations on the MAG receptor side might indeed go along with cognate variations on the ligand side. It will be interesting to learn about structural details of these apparently flexible ligand-receptor interactions.

In sum, our findings support a model of MAG inhibition that is cell-type specific and relies on at least three distinct, but partially redundant, signaling modes. In sensory neurons, NgR1 and NgR2 account only to a minor extent for inhibitory signaling; sialylated components, presumably including the gangliosides GT1b and GD1a, as well as presently unknown factors which exert their inhibitory activity independently from sialylation account for the major effect or can effectively compensate for the loss of the NgR-type MAG receptors. On the other hand, preventing MAG from binding to sialic acid (and thus to gangliosides) or disrupting gangliosides with VCN does not interfere with MAG's inhibitory activity, presumably because NgRs and presently unknown sialylation independent factors can functionally compensate. NgR1 has in fact been demonstrated to mediate MAG inhibition in a VCN-insensitive manner [11], [12]; the same holds true for NgR2, although its preferred way seems to depend on sialic acid binding of MAG [13]. The latter observation is significant because it raises the possibility that the NgR and ganglioside pathways, while distinct with regard to MAG binding, are not necessarily independent from each other. Gangliosides are small lipid molecules which are not likely to transduce signals by their own, but rather depend on specific carrier proteins. NgR1, intriguingly, has recently been identified as GT1b binding protein [39]. As further shown, the GT1b-binding site is spatially distinct from the MAG interaction site [39], [40]. It is thus tempting to speculate that NgR1 forms a dual MAG receptor complex with two distinct binding sites for MAG; an indirect and VCN-sensitive one via the GT1b-binding site and a direct and VCN-insensitive one via its MAG interaction site.

Our data presented herein will help integrate knowledge on the intricacies of MAG inhibition. Detailed information on the underlying mechanisms will be vital for conceiving therapeutic strategies to promote regeneration in the lesioned central and peripheral nervous system.

### Note

While this work was under review, Tessier-Lavigne and colleagues published results showing that PirB, an Ig-like domain containing transmembrane protein, is a functional MAG receptor transducing inhibitory activity in CGN and DRG neurons [41], a finding that is in perfect line with our data presented herein.

## Supporting Information

Figure S1 Activity of VCN on cultured sensory neurons. In order to address the specificity and effectiveness of VCN in our neuronal culture, we treated sensory neurons after plating with 5 mU/ml of VCN and (A) lysed the cells after approx. 20 hours. Immunoblotting of p75NTR, of which there are no reports about sialylation, demonstrates the absence of proteolytic side-effects of VCN. (B) Removal of sialic acid by VCN treatment increases binding of cholera toxin (CT) to mono-sialoganglioside GM1 as revealed by immunocytochemistry. The staining intensity of p75NTR is not affected by VCN treatment. Scale bar 40 µm.
(0.64 MB TIF)

Figure S2 Glycosylation of wildtype MAG and MAG mutants. Wildtype and mutant MAG molecules which were expressed in CHO-K1 clonal lines are differentially glycosylated; importantly, the fully glycosylated form which, in wildtype MAG, migrates at approx. 100 kD, is present in the wildtype and in all mutants (arrow). Treatment of lysates with N-glycosidase F gives rise to an approx. 72 kD immunoreactive band (asterisk) which corresponds to the molecular weight of MAG core protein plus some O-linked sugars [1]. MAG Ig1–3 is of lower molecular weight than the other mutants but is shifted proportionally upon N-glycosidase treatment. Anti-MAG antibody was used to detect wildtype and mutant MAG molecules.
(0.08 MB TIF)

Method S1 (0.03 MB DOC)

Method S2 (0.02 MB DOC)
