# Supplementary figures and images for: Inhibitory Activity of Myelin-Associated Glycoprotein on Sensory Neurons Is Largely Independent of NgR1 and NgR2 and Resides within Ig-Like Domains 4 and 5
Source: PLoS One. 2009 Apr 15;4(4):e5218. doi: 10.1371/journal.pone.0005218 (PMC2666269; doi:10.1371/journal.pone.0005218)

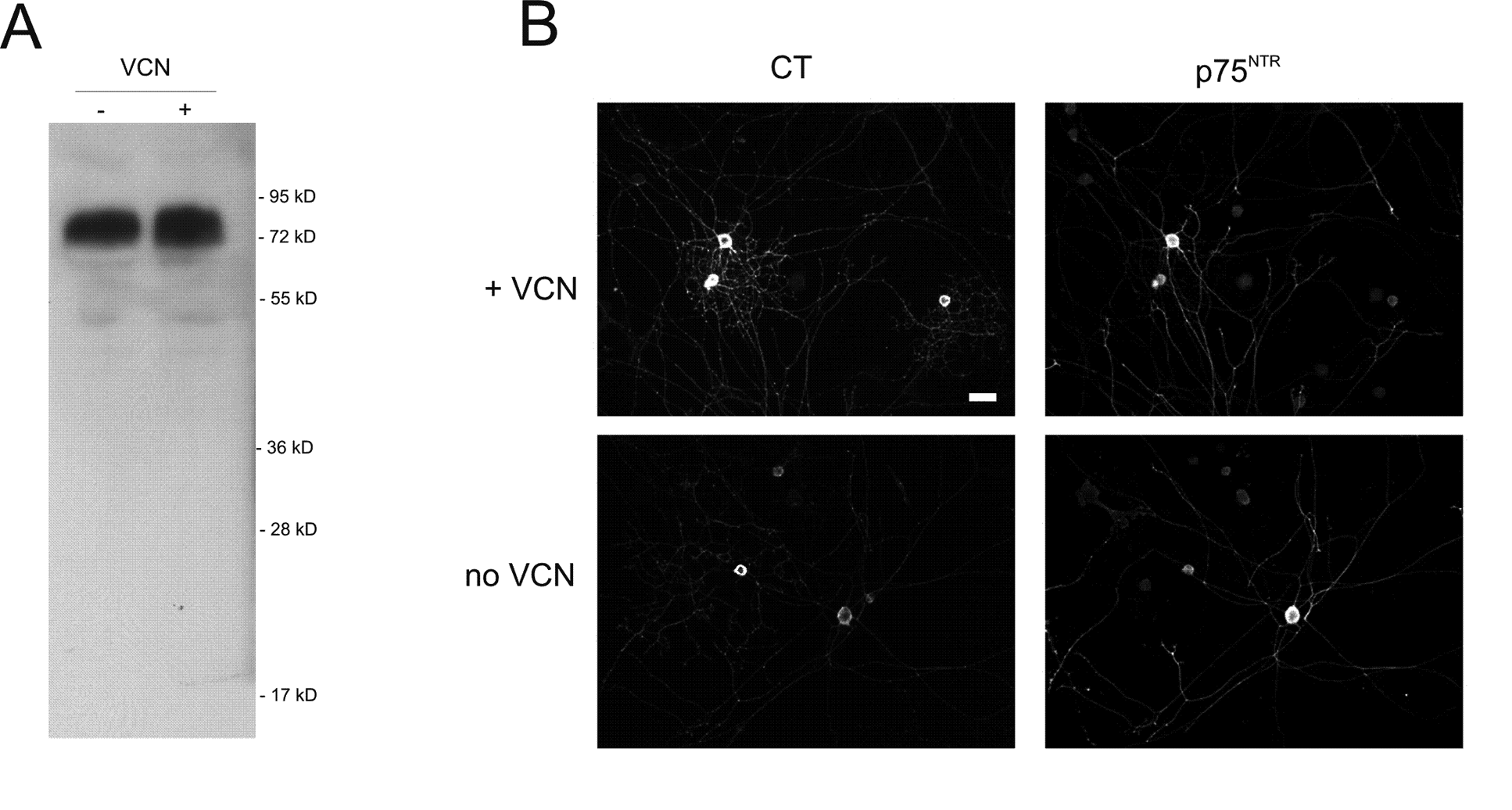

Supplement: Figure S1 — Activity of VCN on cultured sensory neurons. In order to address the specificity and effectiveness of VCN in our neuronal culture, we treated sensory neurons after plating with 5 mU/ml of VCN and (A) lysed the cells after approx. 20 hours. Immunoblotting of p75NTR, of which there are no reports about sialylation, demonstrates the absence of proteolytic side-effects of VCN. (B) Removal of sialic acid by VCN treatment increases binding of cholera toxin (CT) to mono-sialoganglioside GM1 as revealed by immunocytochemistry. The staining intensity of p75NTR is not affected by VCN treatment. Scale bar 40 µm. (0.64 MB TIF) [file pone.0005218.s001.tif]

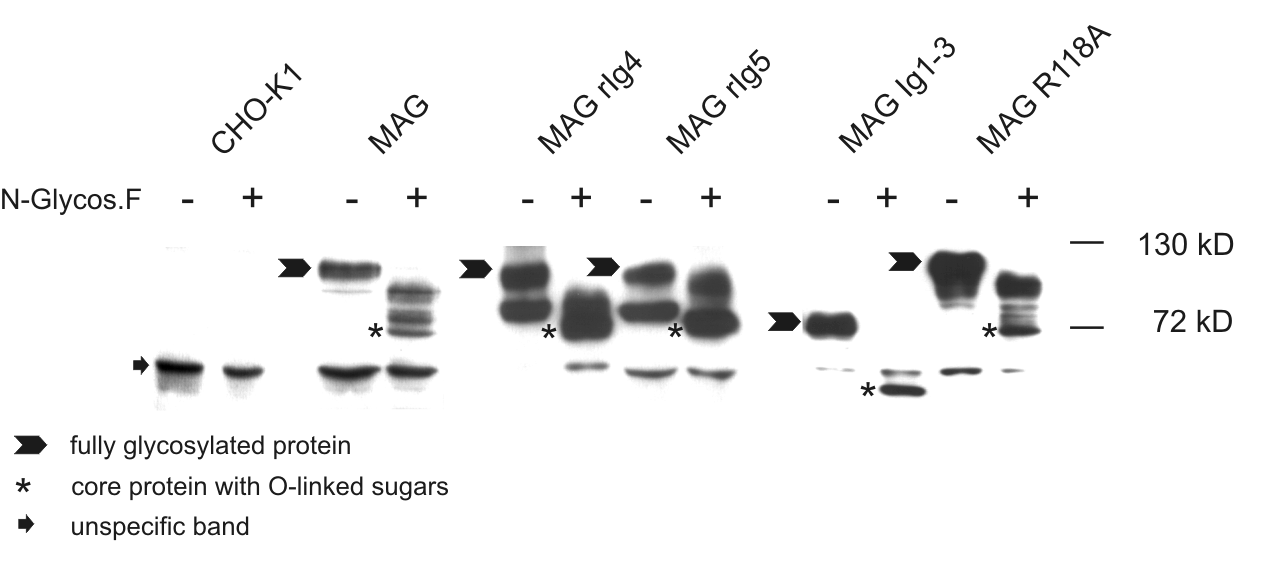

Supplement: Figure S2 — Glycosylation of wildtype MAG and MAG mutants. Wildtype and mutant MAG molecules which were expressed in CHO-K1 clonal lines are differentially glycosylated; importantly, the fully glycosylated form which, in wildtype MAG, migrates at approx. 100 kD, is present in the wildtype and in all mutants (arrow). Treatment of lysates with N-glycosidase F gives rise to an approx. 72 kD immunoreactive band (asterisk) which corresponds to the molecular weight of MAG core protein plus some O-linked sugars [1]. MAG Ig1–3 is of lower molecular weight than the other mutants but is shifted proportionally upon N-glycosidase treatment. Anti-MAG antibody was used to detect wildtype and mutant MAG molecules. (0.08 MB TIF) [file pone.0005218.s002.tif]
