# Supplementary material for: Inhibitory Activity of Myelin-Associated Glycoprotein on Sensory Neurons Is Largely Independent of NgR1 and NgR2 and Resides within Ig-Like Domains 4 and 5
Source: PLoS One. 2009 Apr 15;4(4):e5218. doi: 10.1371/journal.pone.0005218 (PMC2666269; doi:10.1371/journal.pone.0005218)
Supplement: Method S1 — (0.03 MB DOC) [file pone.0005218.s003.doc]

**Method S1: VCN treatment of sensory neurons**

Where indicated Vibrio cholerae neuraminidase was added at 5 mU/ml one hour after plating the neurons. Following an incubation period of approximately 20 hours cells were either lysed for immunoblotting or fixed for immunocytochemistry. Lysis buffer was 50 mM Hepes pH 7.45, 150 mM NaCl, 10% (v/v) glycerol, 1% (v/v) Triton X-100, 10 µg/ml aprotinin, 10 µg/ml leupeptin, 1 mM PMSF. Western Blotting was performed as described in the main text; for detection of p75^NTR^ a polyclonal antiserum was used (1:2000; Promega #G323A), blocking and antibody incubation was in 3.5 % (w/v) skim milk (Merck #1.15363.0500) in TBS/0.1% (v/v) Tween-20; the corresponding HRP-conjugated secondary antibody was goat anti-rabbit IgG (1:20.000; Pierce #31460) diluted in 3.5 % (w/v) skim milk in TBS/0.1% (v/v) Tween-20. For immunocytochemistry cells were washed once and incubated with Choleratoxin Subunit B - Alexa Fluor 488 (Invitrogen, C-22841; 5 µg/ml in 20 mM HEPES, 150 mM NaCl, 10 mM glucose, 0,1 % BSA, pH 7,4) for 15 min at 37°C, prior fixation for 20 min with 4% (w/v) PFA/5% (w/v) sucrose. This was followed by overnight incubation with rabbit polyclonal anti-p75^NTR^ antibody raised against the extracellular domain of rat p75^NTR^ (REX; 1:2000 in 3% BSA/PBS; generously provided by Louis F. Reichardt; (1)) at 4°C. To visualize the p75^NTR^-staining cells were incubated with TRITC-coupled secondary anti-rabbit antibody (2 µg/ml) for one hour at room temperature.

1 Weskamp G and Reichardt LF (1991). Evidence that biological activity of NGF

is mediated through a novel subclass of high affinity receptors. Neuron 6(4):

649-63.
