# Supplementary material for: Inhibitory Activity of Myelin-Associated Glycoprotein on Sensory Neurons Is Largely Independent of NgR1 and NgR2 and Resides within Ig-Like Domains 4 and 5
Source: PLoS One. 2009 Apr 15;4(4):e5218. doi: 10.1371/journal.pone.0005218 (PMC2666269; doi:10.1371/journal.pone.0005218)
Supplement: Method S2 — (0.02 MB DOC) [file pone.0005218.s004.doc]

**Method S2: Deglycosylation of wildtype and mutant MAG**

CHO-K1 clonal cell lines expressing wildtype and mutant MAG were lysed in 100 mM sodium phosphate buffer pH 7.40, 20 mM EDTA, 10% (v/v) glycerol, 1% (v/v) Triton X-100, 0.1% (w/v) SDS, 1% /v/v) ß-mercaptoethanol, 10 µg/ml aprotinin, 10 µg/ml leupeptin, 1 mM PMSF, incubated on ice for 30 min and centrifuged with 21.000 x g at 4°C for 5 min. The supernatant was heated to 65°C for 1 hour and, subsequently, to 95°C for 3 min. Following brief centrifugation the supernatant was mixed with 3 x vol of glycosidase buffer (same as lysis buffer but without glycerol). For approx. 20 µg total protein 1 unit of N-glycosidase F (Roche #11365169001) was added and incubated overnight at 37°C. Immunoblotting was performed using 0.2 µg/ml polyclonal goat anti-MAG (L-20; Santa Cruz #sc-9543).

1 Pedraza L, Frey AB, Hempstead BL, Colman DR and Salzer JL (1991).

Differential expression of MAG isoforms during development. J Neurosci Res

29(2): 141-8.
